# Supplementary figures and images for: Specific Transcriptomic Signatures and Dual Regulation of Steroidogenesis Between Fetal and Adult Mouse Leydig Cells
Source: Front Cell Dev Biol. 2021 Jun 28;9:695546. doi: 10.3389/fcell.2021.695546 (PMC8273516; doi:10.3389/fcell.2021.695546)

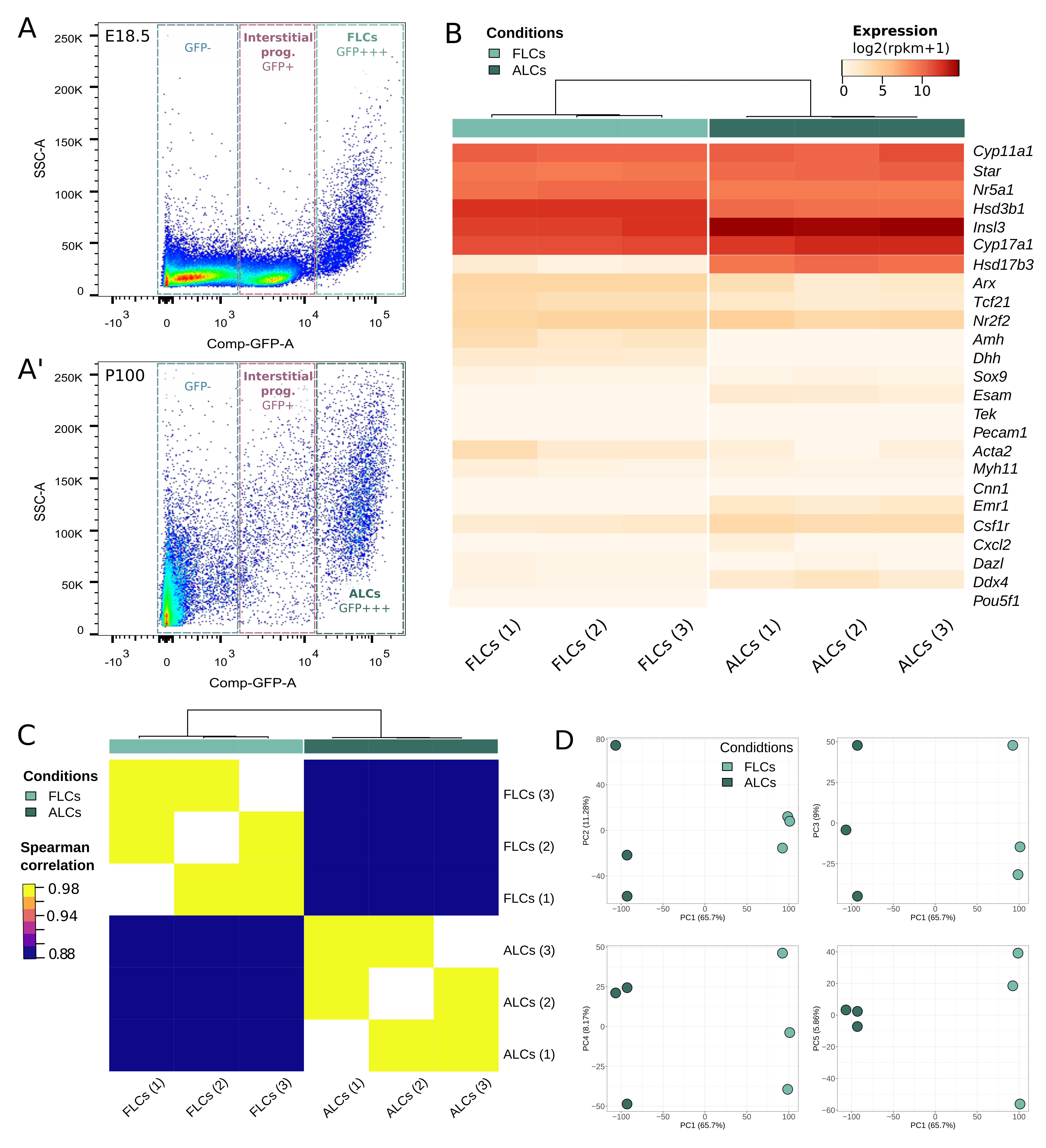

Supplement: Supplementary Figure 1 — (A,A′) Scatter plot showing the selection of Leydig cells (GFP+++) by FACS in E18.5 (A) and in P100 (A′) testis cell suspension. The X-axis corresponds to the GFP florescence level and the Y-axis forward scatter area explaining the granularity of the events. (B) Heatmap showing the normalized scaled expression of selected marker genes in our three replicates at E18.5 (pale green) and in our three replicates at P100 (dark green). The color is representative of the expression level. The gene expression confirmed the purity of the samples in Leydig cells. Cyp11a1, Star, Nr5a1, Hsd3b1, Insl3, Cyp17a1, Hsd17b3, and eGFP: Leydig cells. Cdh5, Pecam1, Acta2, and Rgs5: Endothelial cells. Pdgfra, Tcf21, Wnt5a, and Nr2f2: Interstitial progenitors. Amh, Lhx9, Dhh, and Sox9: Sertoli cells. Pou5f1, Mael, Dadx4, and Dazl: Germ cells. (C) Heatmap showing the Spearman correlation score between the six Leydig cells samples. The score is indicated by the color scale. (D) Principal Component Analysis (PCA) plot where each dot corresponds to a Leydig cells sample. The dots are colored according to the sample type. E18.5: pale green; P100: dark green. [file Image_1.TIFF]

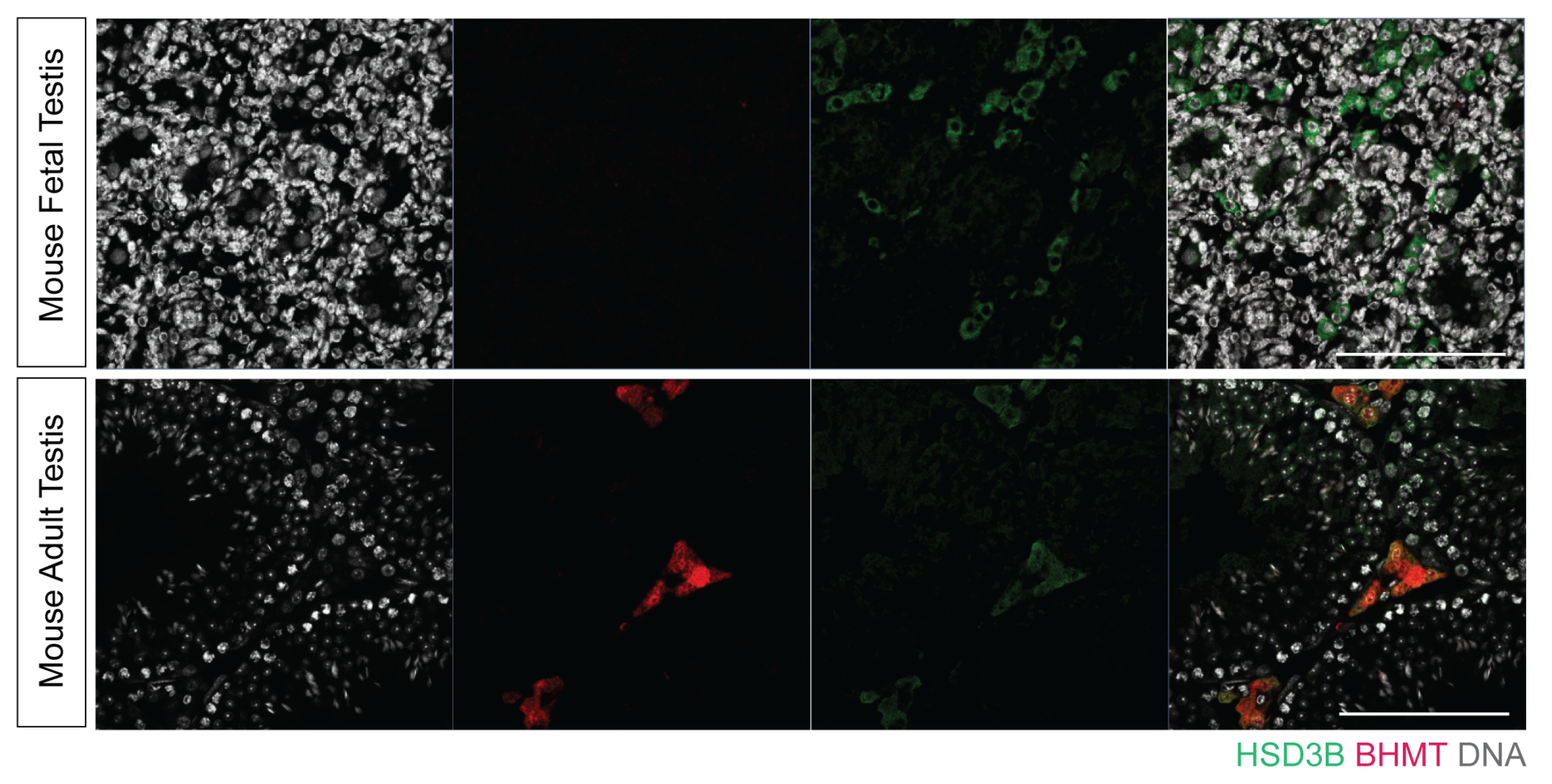

Supplement: Supplementary Figure 2 — Immuno-histochemistry (IHC) staining on mouse fetal and adult testis. The DNA is colored in white, HSD3B labels Leydig cells in green and BHMT is colored in red. The white scale bar corresponds to 50 μm. [file Image_2.TIFF]
